# Associations between long-term adherence to healthy diet and recurrent depressive symptoms in Whitehall II Study

**DOI:** 10.1007/s00394-019-01964-z

**Published:** 2019-04-13

**Authors:** Daisy Recchia, Amaria Baghdadli, Camille Lassale, Eric Brunner, Jean-Michel Verdier, Mika Kivimäki, Tasnime Akbaraly

**Affiliations:** 1 Inserm, U1198, University of Montpellier, Ecole Pratique des Hautes Etudes, PSL Research University, Montpellier, 34095 France; 2 Department of Psychiatry and Autism Resources Centre, University Research and Hospital Center of Montpellier, 291 Avenue du Doyen Gaston Giraud, 34295 Montpellier Cedex 5, France; 3 Centre de Recherche en Épidémiologie et Santé des Populations, U1178, INSERM, Paris, France; 4 Department of Epidemiology and Public Health, University College London, London, WC1E 6BT UK; 5 Department of Behavioural Science and Health, University College London, London, WC1E 6BT UK

**Keywords:** Prospective cohort study, Dietary indices, Depressive symptoms, Alternate Healthy Eating Index-2010, Dietary Approach to Stop Hypertension, Transformed Mediterranean Diet Score

## Abstract

**Purpose:**

We examined whether long-term adherence to three diet quality scores—the Alternative Healthy Eating Index-2010 (AHEI-2010), Dietary Approach to Stop Hypertension (DASH) and  transformed-Mediterranean Diet Score (tMDS), Alternative Healthy Eating Index-2010 (AHEI-2010) and Dietary Approach to Stop Hypertension (DASH) is associated with the risk of recurrent depressive symptoms.

**Methods:**

Analyses were conducted on a sample of 4949 men and women from the Whitehall II study. Diet scores were calculated using data collected from food frequency questionnaires repeated over 11 years of exposure (1991/1993–2002/2004). Recurrence of depressive symptoms was defined when participants reported at least two episodes of depressive symptoms (assessed by Center for Epidemiologic Studies Depression Scale and use of antidepressants) over the four phases of follow-up (2002/04–2015/16).

**Results:**

After adjustment for potential cofounders, higher scores on AHEI-2010, DASH and tMDS at the end of the exposure period were associated with lower risk of recurrent depressive symptoms over the 13-year follow-up. Repeat measures of dietary history showed that participants who maintained a high AHEI-2010 score over the 11-year exposure period had a 19% (OR 0.81, 95% CI 0.65–1.00) lower odds of recurrent depressive symptoms compared to those who maintained a low AHEI score. Participants whose AHEI-2010 score decreased over time had a 1.34-fold increased odds (95% CI 1.02–1.75) of developing recurrent depressive symptoms compared to those maintaining a high AHEI-2010. No robust associations were observed for long-term tMDS and DASH.

**Conclusion:**

Our findings suggest that long-term adherence to healthy diet defined by Alternative Healthy Eating Index-2010 confers protection against recurrent depressive symptoms.

**Electronic supplementary material:**

The online version of this article (10.1007/s00394-019-01964-z) contains supplementary material, which is available to authorized users.

## Introduction

Depression affects over 350 million people worldwide and is considered to be a major contributor to global disability [1]. The annual economic cost of depression in Europe has been estimated at up to €118 billion [counting direct costs (outpatient care, drug cost, hospitalization) and indirect costs due to morbidity and mortality], making depression the most costly brain disorder in Europe [2]. In a context where conventional treatments only address one-third of the disease burden linked to mood [3], it may be crucial to identify modifiable risk factors on which intervention strategies can be based. Amongst those modifiable risk factors, dietary behaviours have raised increased interest in the last few years [4].

Research to date has focused on the associations of specific nutrients like fatty acids [5–9] and B vitamins, such as B6, B9 and B12 [6, 10–12] with depression. Results of those studies are inconclusive and research has increasingly moved from focusing on the effect of isolated nutrients to those of dietary patterns/indices, aiming the consideration of overall effect of diet on depression. Dietary indices measure the quality of a diet by calculating healthy diet scores based on dietary guidelines. Similarities in the recommendations for a healthy diet are a high intake of fruits, vegetables, legumes, whole grains and a low intake of red or processed meats. Differences between dietary indices are the method used to calculate the dietary scores and inclusion of specific food items or nutriments (such as sodium, alcohol, sweet beverages).

Our recent systematic review and meta-analysis of observational studies assessed the association between healthy dietary scores and depressive outcomes and concluded that adhering to a healthy diet appears to confer some protection against depression [13]. Amongst the studied dietary indices, the Mediterranean Diet Score was the most examined index, regarding depressive symptoms/depression and was robustly associated to the incidence of depressive symptoms/depression [13]. Other indices such as the Alternate Healthy Eating Index of 2010 and the Dietary Approach to Stop Hypertension have also been studied in relation to depressive symptoms/depression [13]. However, longitudinal studies assessing the long-term impact of dietary habits on depressive disorders remain scarce. Even though some studies provide evidence that the quality of diet is associated with depressive symptoms/disorders, they also suggest that more prospective studies are needed to confirm this association [14].

Temporality is a major issue in the assessment of the healthy diet-depression relation, especially in a public health context and dietary guidelines to reduce the risk of depressive symptoms.

Accordingly, we aimed to examine whether long-term adherence to the existing dietary recommendations,  assessed by the Alternate Healthy Eating Index using the 2010 criteria (AHEI-2010), ﻿the Dietary Approach to Stop Hypertension (DASH) and the transformed Mediterranean Diet Score (tMDS), are associated with the recurrence of depressive symptoms assessed over 13 years in a British cohort of men and women.

## Methods

### Study population

Analyses were conducted using data from Whitehall II, prospective cohort study including 10,308 participants (6895 men, 3413 women) [15]. In 1985/1988 all persons aged 35–55 years working in 20 London-based departments were invited to participate by letter, and 73% agreed. Since baseline, follow-up clinical examinations have taken place approximately every 5 years: 1991/1993 (*n* = 8815), 1997/1999 (*n* = 7870), 2002/2004 (*n* = 6967), 2007/2009 (*n* = 6761), 2012/2013 (*n* = 6318) and 2015/2016 (*n* = 5632). Written informed consent was obtained after a thorough explanation of the study to each of the participants; the University College London ethics committee approved the study.

**Fig. 1:**
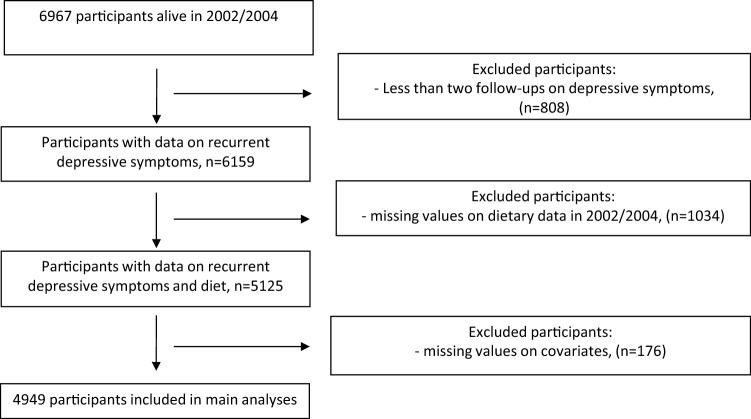
Flow chart diagram mapping the selection of participants

Analyses were restricted to participants with complete data on dietary assessment and covariates in 2002–2004. Also, participants with less than two follow-ups out of four phases (2002/2004, 2007/2009, 2012/2013 and 2015/2016) were excluded. 4949 participants were thus included in main analyses as detailed in the flow-chart diagram (Fig. 1).

### Depressive symptoms (DepS) assessed over a 13 year period (2002/2004–2015/2016)

Depressive symptoms (DepS) were assessed using the Center for Epidemiologic Studies Depression Scale (CES-D) [16]. This scale is composed of 20 items matching DepS, the frequency of DepS was evaluated using a four-point scale. The scale ranges from ‘less than once a week’ to ‘5–7 days a week’. Participants self-reported details of current medications use (generic name, brand name, or both); these were subsequently coded using the British National Formulary to determine antidepressant use. We defined cases of DepS as participants with a CES-D score ≥ 16 or those treated by antidepressants at four phases (2002/2004, 2007/2009, 2012/2013 and 2015/2016).

Recurrence of DepS was defined as presenting DepS at two, three or all the four phases of follow-up, while non-recurrent cases were defined when participants reported one or no DepS episodes over the 13 years of follow-up.

### Assessment of dietary intake

Dietary intake was assessed in 1991/1993, 1997/1999 and 2002/2004 with a semi-quantitative food frequency questionnaire (FFQ) [17, 18] based on one used in US Nurses' Health study [19]. Participants were asked how often, on average, they had consumed a common unit or portion size of each food item during the previous year. A nine-point scale was used to assess the frequency at which food items were consumed. The scale ranges from ‘never or less than once per month’ to ‘six or more times per day’. Daily food consumption was computed for each participant based on the FFQ. Nutrient intakes were calculated by multiplying the consumption frequency for each food by its nutrient content (for specified portions) and then summing nutrient contributions from all foods. This was done using the computerized system developed for the Whitehall II dietary data and based on the 4th and 5th editions of McCance and Widdowson’s The Composition of Foods and supplementary tables as detailled elsewhere [20].

### Diet indices Scores

Three dietary scores assessing the overall diet quality were derived from the FFQ data at all three phases:

*The Alternate Healthy Eating Index of 2010 (AHEI*-*2010)* [21] is based on 11 components, six components for which the highest intakes are considered to be ideal (vegetables, fruits, whole grains, nuts and legumes, long chain omega-3 fats (DHA and EPA) and polyunsaturated fatty acids), one component for which moderate intake was supposed to be ideal (alcohol), and four components for which avoidance or lowest intake are considered to be ideal (sugar-sweetened drinks and fruit juice, red and processed meat, trans fat, and sodium). Each component is given a minimal score of 0 and a maximal score of 10, with intermediate values scored proportionally. All the component scores are summed to obtain a total AHEI-2010 score, which ranges from 0 to 110, with a higher score representing a healthier diet.

*The Dietary Approach to Stop Hypertension (DASH)* [22] is based on seven components, four components for which the highest intakes are considered to be ideal (vegetables, fruits, whole grains and legumes) and three components for which avoidance or lowest intake are considered to be ideal (transformed and red meat, sweet beverages and sodium). Each component is given a minimal score of 1 and a maximal score of 5, with intermediate values scored proportionally. All component scores are summed to obtain a total DASH score, which ranges from 5 to 40, with a higher score representing a healthier diet.

*Transformed Mediterranean Diet Score (tMDS) * [23] is based on 9 components, five components for which higher intakes are considered to be ideal (vegetables, fruits, whole grains, fish, legumes, mono unsaturated fatty acids/saturated fatty acids ratio) and thee components for which lowest intake are considered to be ideal (meat, dairy products and alcohol). Each component is given a minimal score of 0 and a maximal score of 1 according to population’s median components’ consumption. All component scores are summed to obtain a total tMDS score, which ranges from 0 to 9, with a higher score representing a healthier diet.

To represent long-term dietary intake and to reduce measurement errors, we calculated the cumulative average of the diet scores over 11-years of exposure period, using the repeated measures of dietary intakes in 1991/1993, 1997/1999 and 2002/2004. To analyse the association of 11 year change in the diet scores with recurrent DepS, scores assessed in 1991/1993 and 2002/2004 were categorized as high or low according to the median value of diet scores in 1991/1993 (58 for AHEI-2010, 24 for DASH and 5 for tMDS). Four categories were then defined: participants who maintained a high score (both scores of 1991/1993 and 2002/2004 ≥ median score), those who maintained a low score (both scores of 1991/1993 and 2002/2004 < median score), participants who improved their diet score (score of 1991/1993 < median score and score of 2002/2004 < median score) and those who decreased their diet score (score of 1991/1993 ≥ median score and score of 2002/2004 < median score).

### Covariates assessed in 2002/2004

Three types of covariates were considered, socio-demographic factors, health behaviours and health status factors and were derived from the questionnaires completed by the participants and from measures assessed during the physical examination in 2002/2004 (measures of systolic and diastolic blood pressures, weight, height, fasting glucose and HDL-cholesterol assessed from fasting blood samples taken from participants).

Socio-demographic data consisted of sex, age, ethnicity (White/South Asian/Black), marital status (married or cohabiting vs. single/divorced/widowed), and socio-economic status (SES) based on occupational position, categorized into three groups: high (administrative), intermediate (professional or executive) and low (clerical or support) [15].

Health behaviours included smoking (classified as “current smoker” or “noncurrent smoker” (including former smokers)), alcohol consumption (classified as “no alcohol consumption in the previous week”, “moderate alcohol consumption” (1–14 units/week in women and 1–21 units/week in men), and “heavy drinkers” (15 + units in women and 21 + units in men)), total energy intake (estimated from the FFQ) and physical activity (classified as “active” (> 2.5 h per week of moderate physical activity or > 1 h per week of vigorous physical activity), “inactive” (< 1 h per week of moderate physical activity and < 1 h per week of vigorous physical activity), or “moderately active” (if neither active nor inactive)) [24]. Details about the collection of this data are to be found elsewhere [25].

Health status factors consisted in prevalent coronary heart disease (CHD) (denoted by clinically verified non-fatal myocardial infarction or definite angina); hypertension (defined by systolic/diastolic blood pressure ≥ 140/90 mm Hg, respectively, or use of antihypertensive drugs); type 2 diabetes (diagnosed according to the WHO definition); serum high-density lipoprotein (HDL) cholesterol measured in mmol/L, body mass index (kg/m^2^) and cognitive impairment defined by a score ≤ 27 in the Mini Mental State Examination (MMSE) [26]. All health status factors added to the statistical models were used as binary categorical variables (yes/no) except for the HDL cholesterol (mmol/L) and the BMI (kg/m^2^) which were analysed as continuous variables.

### Statistical analysis

Characteristics of participants according to the number of depressive episodes, participants included in this study versus excluded participants and participants’ characteristics as a function of the three diet scores were compared. Chi-squared tests were used for categorical variables and ANOVA tests or Student *t* tests were used for continuous variables.

Characteristics associated with the number of depressive episodes or with diet scores were selected to enter the multivariable models as covariates with a potential role of confounder.

To assess whether diet quality scores in 2002/2004 or over 11-years of exposure period (between 1991/1993 and 2002/2004) was associated with recurrence of DepS over the 13-year of follow-up, we performed logistic regression models. Diet scores at in 2002/2004 or their 11-year cumulative average scores (between 1991/1993 and 2002/2004) were analysed as a continuous standardized variable using *z*-score [mean = 0, standard deviation (SD) = 1] allowing the odds of recurrent and non-recurrent DepS per 1 SD increment of the considered dietary score to be estimated. Change in diet was assessed as categorical variables. Three levels of adjustment have been considered (Model 0 was the non-adjusted model). Model 1 was first adjusted for basic socio-demographic variable including age, sex and ethnicity) and total energy intake. The Model 1 was then additionally adjusted for other socio-demographic variable (occupational grade, marital status) and health behaviour factors (smoking behaviour, physical activity, alcohol consumption—only for the DASH index), (Model 2) and finally for health status variables (cardiovascular disease, type 2 diabetes, hypertension, HDL-cholesterol, body mass index and cognitive impairment), (Model 3). Adjustments on alcohol consumption were only added for the DASH index since alcohol is part of AHEI-2010 and tMDS scores construction.

For sensitivity analyses associations between diet scores and recurrent DepS were repeated excluding participants who had DepS in 1991/1993 and in 1997/1999, assessed by the General Health Questionnaire (GHQ) [27]. GHQ captured common mental disorders and included the four-item depression subscale. All items were scored from 0 to 3 and then summed; cut-off points of 4 out of 12 were used to identify depression cases.

To examine effect modification, interactions between main covariates and diet scores regarding DepS have been tested.

## Results

The present analyses included 4949 participants and excluded 2018 participants. The participants were more likely to be younger, men, white, married, with high socio-economic status, to have healthy behaviours (non-smoker, high level of physical activity, moderate alcohol consumption) and to be healthier in general. Included participants were also less likely to have recurrent DepS. They did not differ from the excluded participants in terms of dietary scores (Supplementary Table A).

Over the 13-years of follow-up, 658 participants presented recurrent DepS (13.3%), 731 had DepS in 1 out of the 4 phases (14.8%), while 3560 of the sample did not have DepS at either phase (71.9%). Table 1 presents the characteristics of participants according to the number of depressive episodes. Participants with recurrent DepS were more likely to be women (with 18.3% of women having recurrent DepS versus 11.4% men), South Asian with low SES, living alone, current smoker, alcohol abstainers, physically inactive, with type 2 diabetes and cognitive impairment.

**Table 1:** Comparison of Whitehall II participants’ characteristics as a function of the number of depressive symptoms episodes over the 13 years follow-up

Characteristics of participants	Number of depressive symptoms episodes (*n* = 4949)			
	0 (*n* = 3560)	1 (*n* = 731)	≥ 2 (*n* = 658)	
	%/Mean ± SD	%/Mean ± SD	%/Mean ± SD	*p*^d^
Socio-demographic factors				
Sex				
Men	76.7	64.0	62.8	< 0.001
Women	23.3	36.0	37.2	
Age (years)	61.1 ± 5.9	60.8 ± 6.0	61 ± 6.1	0.46
Ethnicity				
White	96.1	91.1	92.1	< 0.001
South Asian	2.5	6.7	6.4	
Black	1.4	2.2	1.5	
Marital status				
Living alone	20.1	28.6	31.8	< 0.001
Married	79.9	71.4	68.2	
Socio-economic status				
High	53.0	41.0	36.6	< 0.001
Intermediate	39.9	45.8	49.7	
Low	7.2	13.1	13.7	
Health behaviour factors				
Smoking habits				
Never/ex	93.8	91.5	90.4	< 0.001
Smoking	6.2	8.5	9.6	
Alcohol intake				
No	12.9	17.9	20.7	< 0.001
Moderate	67.1	62.0	60.2	
Heavy	20.0	20.1	19.2	
Physical activity				
Inactive	22.0	28.3	29.5	< 0.001
Moderate	16.2	16.4	19.5	
Active	61.8	55.3	51.1	
Total energy intake (kcal/day)	2156.6 ± 572.5	2135.9 ± 611.5	2150.7 ± 615.9	0.68
Health status factors				
Heart disease				
No	92.0	89.9	90.0	0.07
Yes	8.0	10.1	10.0	
Hypertension				
No	61.8	63.2	61.7	0.77
Yes	38.2	36.8	38.3	
Type 2 diabetes				
No	91.8	88.4	90.7	0.01
Yes	8.2	11.6	9.3	
HDL cholesterol (mmol/l)	1.6 ± 0.4	1.60 ± 0.4	1.58 ± 0.4	0.15
Body Mass Index (kg/m^2^)	26.5 ± 4.1	26.67 ± 4.4	26.77 ± 4.5	0.24
Cognitive impairment				
No	88.2	86.2	82.7	< 0.001
Yes	11.8	13.8	17.3	
Diet quality scores				
AHEI-2010^a^	58.7 ± 10.7	58.3 ± 11.2	57.4 ± 11.7	0.02
DASH^b^	24.2 ± 4.7	24.1 ± 5.0	23.7 ± 4.8	0.04
tMDS^c^	4.6 ± 1.6	4.5 ± 1.7	4.4 ± 1.7	< 0.001
11-year cumulative average diet score				
AHEI-2010^a^	58.4 ± 9.0	57.9 ± 9.2	57.2 ± 9.7	0.01
DASH^b^	24.2 ± 4.1	24.1 ± 4.3	23.8 ± 4.3	0.08
tMDS^c^	4.6 ± 1.3	4.6 ± 1.4	4.5 ± 1.3	0.10

Values are percentage or mean ± standard deviation

^a^*AHEI-2010* Alternate Healthy Eating Index of 2010

^b^*DASH* Dietary Approach to Stop Hypertension

^c^*tMDS* transformed Mediterranean Diet Score

^d^*p* values are based on *X*^2^ test or ANOVA test

Mean diet quality scores in 2002/2004 were 58.5 (SD 10.9) for the AHEI-2010, 24.1 (SD 4.8) for the DASH and 4.6 (SD 1.7) for the tMDS. Correlation between the three diet scores varied between 0.47 (tMDS AND DASH), 0.57 (AHEI-2010 and tMDS) and 0.74 (AHEI-2010 and DASH) and were all statistically significant. Characteristics of participants according to diet scores have been described in Supplementary Table B.

**Fig. 2:**
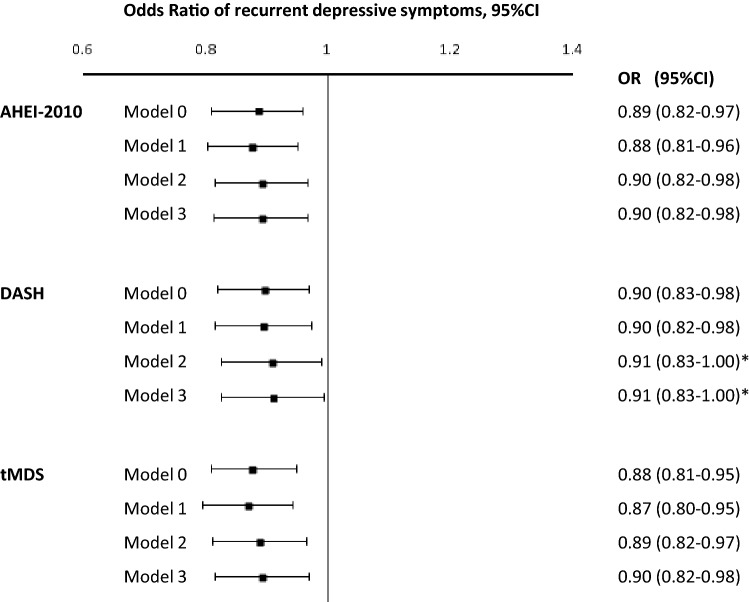
Odds ratios (95% confidence intervals) for the association between diet scores (2002/04) and the recurrent depressive symptoms (DepS) over 13 years of follow-up in 4949 Whitehall II participants. *Only for 4602 non-smoking Whitehall II participants; *AHEI-2010* Alternate Healthy Eating Index of 2010, *DASH* Dietary Approach to Stop Hypertension, *tMDS* transformed Mediterranean Diet Score; Logistic regression models were performed. *Model 0* non adjusted. Model 1: adjusted for age, sex, ethnicity and total energy intake, *Model 2* model 1 plus adjustment for occupational grade, marital status, smoking behaviour, physical activity, alcohol consumption (for the DASH index only), *Model 3* model 2 plus adjustment for type 2 diabetes, CHD, hypertension, HDL-cholesterol, body mass index and cognitive impairment

Results of logistic regression models adjusted for sex, age, ethnicity and total energy intakes examining associations between the three diet quality scores assessed in 2002/2004 and the recurrent DepS between 2002/2004 and 2015/2016 showed that higher diet scores (healthier diet quality) were associated with lower odds of recurrent DepS (for the AHEI-2010, OR 0.88, 95% CI 0.81–0.96; for the DASH, OR 0.90, 95% CI 0.82–0.98; for the tMDS, OR 0.87, 95% CI 0.80–0.95). Interactions tested between the three dietary indices and main covariates were not statistically significant, except for one interaction found between the DASH and the smoking status of participants. Thus, results for DASH score (Model 2 and 3) are from now on only presented for non-smoking Whitehall II participants (*n* = 4602). Results of the multivariable analyses assessing the association between dietary scores in 2002/2004 and the 13-year recurrence of DepS are illustrated in Fig. 2. Whatever the level of adjustment, higher scores of AHEI-2010, DASH and tMDS were associated with a reduced odds of recurrent DepS, in fully adjusted models for each increment of 1 standard deviation of the diet score, odds ratio were as follows: OR 0.90, 95% CI 0.83–0.98; OR 0.91, 95% CI 0.83–1.00; OR 0.90, 95% CI 0.82–0.98 for the AHEI-2010, the DASH and the tMDS, respectively (Fig. 2).

**Fig. 3:**
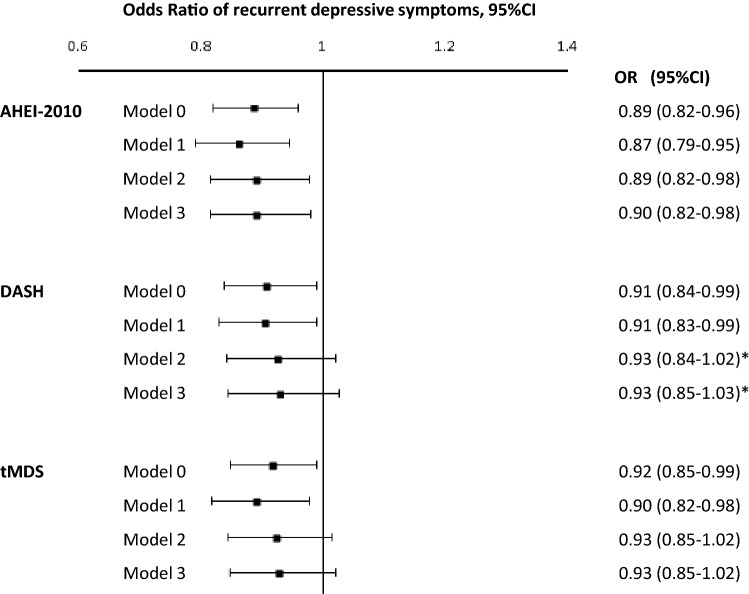
Odds ratios (95% confidence intervals) for the association between cumulative average of diet scores (1991/94–2002/04) and the recurrent depressive symptoms (DepS) over 13 years of follow-up in 4949 Whitehall II participants. *Only for 4602 non-smoking Whitehall II participants. *AHEI-2010* Alternate Healthy Eating Index of 2010, *DASH* Dietary Approach to Stop Hypertension, *tMDS* transformed Mediterranean Diet Score, *Model 0* non adjusted, *Model 1* adjusted for age, sex, ethnicity and total energy intake, *Model 2* model 1 plus adjustment for occupational grade, marital status, smoking behaviour, physical activity, alcohol consumption (for the DASH index only), *Model 3* model 2 plus adjustment for type 2 diabetes, CHD, hypertension, HDL-cholesterol, body mass index and cognitive impairment

To analyse the association between long-term diet quality and the recurrence of DepS, we repeated the analyses by considering the cumulative average of diet scores over 11-year of exposure period. In analyses adjusted for age, sex, ethnicity and total energy intake, higher cumulative average diet quality scores were associated with reduced odds of recurrent DepS. However, after taking into account the health behaviour confounders (smoking status, alcohol consumption (for the DASH index only), physical activity and energy intake) and health status factors (heart disease, hypertension, type 2 diabetes, HDL cholesterol, body mass index, cognitive impairment) associations remained statistically significant only for the AHEI-2010 (Fig. 3, for the AHEI-2010, OR 0.90, 95% CI 0.82–0.98; for the DASH, OR 0.93, 95% CI 0.85–1.03; for the tMDS, OR 0.93, 95% CI 0.85–1.02).

Further analyses (Table 2) showed that participants who maintained a high score of AHEI-2010 (scores in 1991/1993 and 2002/2004 ≥ 58 points) over the 11-year measurement period had 19% (OR 0.81, 95% CI 0.65–1.00) lower odds of recurrent DepS compared to participants who maintained a low AHEI-2010 score (scores in 1991/1993 and 2002/2004 < 58 points). Similarly those who improved their AHEI-2010 score (score in 1991/1993 < 58 points and scores in 2002/2004 score ≥ 58 points) had 16% (OR 0.84; 95% CI 0.65–1.09) lower odds of recurrent DepS compared to participants who maintained a low AHEI-2010 score, however, the association did not reach the statistical significance. Conversely participants whose AHEI-2010 score decreased over time (score in 1991/1993 ≥ 58 and scores in 2002/2004 < 58 points) increased by 34% their odds of developing recurrent DepS compared to participants who maintained high AHEI-2010 score over the 11-year exposure period (OR: 1.34, 95% CI 1.02–1.75). These analyses were performed for the DASH and the tMDS (Supplementary tables C and D) with no significant associations observed for both indices in full adjusted model.

**Table 2:** Odds ratios (95% confidence intervals) for the association between 11-year change in AHEI-2010 score between 1991/1993 and 2002/2004 and the recurrent depressive symptoms (DepS) over 13 years of follow-up

11-year change in AHEI-2010^a^	*n*	OR	95% CI
Maintaining a high AHEI-2010^a^ score	1584	0.81	0.65–1.00
vs. maintaining a low AHEI-2010^a^ score	1651	1	Ref.
Improving AHEI-2010^a^ score	788	0.84	0.65–1.09
vs. maintaining a low AHEI-2010^a^ score	1651	1	Ref.
Decreasing AHEI-2010^a^ score	681	1.34	1.02–1.75
vs. maintaining a high AHEI-2010^a^ score	1584	1	rEf.

Models were adjusted for age, sex, ethnicity, marital status, socio-economic status, smoking habits, physical activity, total energy intake, heart diseases, hypertension, type 2 diabetes, HDL cholesterol, Body Mass Index, cognitive impairment

Maintaining a high score: both scores of 1991/94 and 2002/04 ≥ median score (58)

Maintaining a low score: both scores of 1991/94 and 2002/04 < median score (58)

Improving score: score of 1991/94 < median score (58) and score of 2002/04 ≥ median score (58)

Decreasing score: score of 1991/94 ≥ median score (58) and score of 2002/04 < median score (58)

^a^*AHEI-2010* Alternate Healthy Eating Index of 2010

### Sensitivity analyses

In sensitivity analysis, associations between diet scores and recurrent DepS were repeated excluding participants who had DepS in 1991/1993 and in 1997/1999 (*n* = 96). DepS were assessed in 1991/1993 and in 1997/1999 using the GHQ depression subscale. Results showed that the associations between all three dietary scores in 2002/2004 remained significantly associated with recurrent DepS. In fully adjusted models for each increment of 1 standard deviation of the diet score, odds ratio were as follows: OR 0.90, 95% CI 0.82–0.98; OR 0.90, 95% CI 0.83–0.99; OR 0.90, 95% CI 0.83–0.99 for the AHEI-2010, the DASH and the tMDS, respectively.

## Discussion

Findings from serial measurements of 4949 participants over 24 years showed that higher long term diet quality score measured during adult life was associated with a reduced risk of recurrent DepS assessed over 13-year of follow-up. Amongst the three diet quality scores assessed, the Alternative Healthy Eating Index-2010 was the most robustly associated with recurrent DepS. Our analyses showed that participants who maintained over a 11-year time period a high adherence to dietary recommendations provided by the AHEI-2010 had approximately 10% lower odds of future recurrent DepS compared to those who maintained low adherence to AHEI-2010. In contrast, participants who worsened the quality of their diet according to AHEI-2010 guidelines had about 30% increased odds of developing recurrent DepS. The associations we reported were independent of sociodemographic and other life habits factors as well as cardio-metabolic risk factors and cognitive impairment.

Literature on prospective associations between overall diet and depression focuses primarily on the impact of the Mediterranean Diet on incident DepS [13]. Our results on the associations between dietary scores and recurrent DepS further support the relation between a Mediterranean diet and depressive outcomes. This study is, however, the first to show that adherence to the DASH is also associated to recurrent DepS and our results are consistent with existing observational studies showing significant associations between DASH scores and depression [28–30]. The importance of considering the impact of dietary electrolytes on mood disorders points out in these studies, a low-sodium diet seems for instance to be associated with greater fatigue compared with a weight reduction or usual diet [31].

For the AHEI, our previous analyses carried out on Whitehall participants showed that the preceding version of the AHEI was associated with the recurrence of DepS assessed over 5 years [32]. This association was reported in women but not in men. However, this sex-specific association was not explained by baseline covariates and the possibility that the study was underpowered remained plausible. In the present analysis, we assessed for the first time whether the updated criteria of the AHEI—the AHEI-2010—was associated with the recurrence of DepS and we reported a significant and robust association with no sex differences.

All three dietary indices showed some similarities as reflected by the high correlations coefficients between their scores. Despite higher correlation between AHEI-2010 and DASH compared to AHEI-2010 and tMDS, analyses of the association between diet scores at baseline and recurrent DepS showed closer estimates of the AHEI-2010 and tMDS *z*-scores associations with recurrent DepS compared to AHEI-2010 and DASH *z*-scores—recurrent DepS associations. While sharing common components, one explanation might be related to the fact that both tMDS and AHEI-2010 account for the alcohol consumption while the DASH did not. Further studies need to quantify the relative importance of alcohol component compared to other components of a diet in regards to depression.

The assessment of the long term adherence to healthy guidelines provided by the three indices and their association with recurrence of DepS constitutes novel findings. The reasons we observed an association with the AHEI-2010 but not with the two other dietary indices regarding the long term impact of diet on depressive outcomes remains unclear. One possibility might be that the scoring system of most components of the AHEI-2010 is based on fixed cut-off while the scoring method of the tMDS is based on the binary attribution of 0 or 1 point according to population medians of components’ consumption. Another possibility is that the sodium and the *trans* fatty acids intakes taken into account in the AHEI-20110 score but not in the tMDS play a differential role on the observed associations assessing the long term impact of diet on the recurrence of DepS. Finally, it is also possible that in this specific population, the AHEI-2010 score is more adapted than the tMDS or the DASH. However, at this stage, additional studies are needed to replicate our findings and further identify the most adapted dietary recommendations for each population regarding onset or recurrence of DepS.

Limitations of the present findings include, firstly, the use of a semi-quantitative FFQ (covering 127 specific food items) to assess the dietary intakes, since it is considered to be less precise than other dietary assessment methods such as dietary records. Specifically, estimation of sodium intake considered in the DASH and the AHEI-2010 scores was obtained by nutrient analysis of food intake collected by the FFQ and did not account for salt added during cooking or to prepared food. Nevertheless, nutrient intake estimated by this FFQ has previously been shown to be well correlated with biomarkers concentrations and intake estimates from generally more accurate 7 day diary [17]. A second limitation of this study is the use of the CES-D to assess DepS. Even though the CES-D scale has been shown to be a reliable and valid measurement tool [33], the repeated measurements of CES-D may not capture the severity or the chronicity of DepS. We sought to take into account this limitation by considering recurrent DepS defined as participants with a CES-D DepS episode at least 2 out of 4 follow-up phases. However, our results cannot be extended to major depression. Another limitation is the bi-directional aspect of the diet depression association. It is hard to conclude to a direction of this association, even with the prospective design of this study. To take into account this limitation, we conducted sensitivity analysis, excluding participants who have previously been considered depressive. Finally, a third drawback concerns the generability of our findings. As Whitehall II participants consist mainly of white, office-based civil servants, which is not fully representative of the British general population. In addition, some bias owing to selective retention of participants is possible as we found socioeconomic status, number of DepS episodes and in a less extent diet indices scores to be associated with the likelihood of being included in the analyses. If anything, this could contribute to an underestimation of the association between healthy dietary scores and recurrent DepS on the account of the overrepresentation of individuals with no recurrent DepS episode who follow a health-conscious diet.

Strengths of this study are, first, its large sample size (nearly 5000 participants) allowing us to assess the associations between diet and recurrent DepS considering 658 cases with recurrent DepS. Second the use of three different diet indices that allows to compare different diet styles, applicable on a British population. Third, the prospective design of this study with 13-year follow up on DepS and an 11-year of exposure period for dietary assessment makes possible to study the longitudinal effect of diet on DepS.

In conclusion, this study provides evidence on the association between long-term adherence to dietary guidelines as those provided by the AHEI-2010 and lower risk of future recurrent DepS over adult life. The use of AHEI-2010 was a definite strength, its fixed cut-offs provides some clear dietary guidelines to the clinicians to assess dietary behaviours and provide a good tool for dietary counselling to patients. Our results add support to the existing evidence suggesting the importance of diet in mental health care [34] and prevention [4]. To go further, the efficiency of dietary improvement intervention based on the AHEI-2010 guidelines on depression outcomes need to be tested.

## Electronic supplementary material

Below is the link to the electronic supplementary material.

Supplementary material 1 (DOCX 26 kb)
