# Supplementary material for: Associations between long-term adherence to healthy diet and recurrent depressive symptoms in Whitehall II Study
Source: Eur J Nutr. 2019 Apr 13;59(3):1031–41. doi: 10.1007/s00394-019-01964-z (PMC7098924; doi:10.1007/s00394-019-01964-z)
Supplement: Supplementary file 1 — Supplementary material 1 (DOCX 26 kb) [file 394_2019_1964_MOESM1_ESM.docx]

**European Journal of Nutrition**

**Associations between long-term adherence to healthy diet and recurrent depressive symptoms in Whitehall II Study**

**Daisy Recchia MSc^1 ●^ Amaria Baghdadli MD PhD^2,3^ ^●^ Camille Lassale PhD^4,5 ●^ Eric Brunner PhD^4^ ^●^ Jean-Michel Verdier PhD^1^ ^●^ Mika Kivimäki PhD, FMedSci^4^ ^●^ Tasnime Akbaraly PhD^1,2,4^**

**Authors’ affiliations** :

^1^ MMDN, University of Montpellier, EPHE, INSERM, U1198, PSL Research University, Montpellier F-34095, France

^2^ Department of Psychiatry & Autism Resources Centre, CHRU de Montpellier F-34000, France

^3^ Centre de Recherche en Épidémiologie et Santé des Populations, U1178, INSERM, Paris, France

^4^ Department of Epidemiology and Public Health, University College London, London WC1E 6BT, United Kingdom

^5^ Department of Behavioural Science and Health, University College London, London WC1E 6BT, United Kingdom

**Corresponding author** :

Tasnime N. Akbaraly

Mécanismes Moléculaires dans les Démences Neurodégénératives - **Université Montpellier**Place Eugène Bataillon CC105
34095 Montpellier cedex 5

Phone : + 33 (0)4 67 14 33 86
Fax : + 33 (0)4 67 14 33 86

E-mail: [tasnime.akbaraly@inserm.fr](mailto:tasnime.akbaraly@inserm.fr)

Correspondence and reprint requests should be addressed to the corresponding author

**Supplementary Table A.** Comparison of included/excluded Whitehall II participants’ characteristics

| **Characteristics of participants** | | **Included (n=4949)** | **Excluded (n=2018)** |  |
| --- | --- | --- | --- | --- |
|  | | **% / Mean ± SD** | **% / Mean ± SD** | **p ^d^** |
| **Socio-demographic factors** | |  |  |  |
| Sex | Men | 73.0 | 63.5 | <.001 |
|  | Women | 27.0 | 36.5 |  |
| Age (years) |  | 61.0 ± 5.9 | 61.8 ± 6.2 | 0.03 |
| Ethnicity | White | 94.8 | 87.1 | <.001 |
|  | South Asian | 3.7 | 7.8 |  |
|  | Black | 1.5 | 5.1 |  |
| Marital Status | Living alone | 22.9 | 28.9 | <.001 |
|  | Married | 77.1 | 71.1 |  |
| Socio-economic status | High | 49.0 | 35.5 | <.001 |
|  | Intermediate | 42.1 | 46.8 |  |
|  | Low | 8.9 | 17.7 |  |
| **Health behaviour factors** | |  |  |  |
| Smoking habits | Never/ex | 93.0 | 88.1 | <.001 |
|  | Smoking | 7.0 | 11.9 |  |
| Alcohol consumption | No | 14.7 | 25.0 | <.001 |
|  | Moderate | 65.4 | 56.7 |  |
|  | Heavy | 19.9 | 18.3 |  |
| Physical activity | Inactive | 23.9 | 32.8 | <.001 |
|  | Moderate | 16.7 | 19.2 |  |
|  | Active | 59.4 | 48.0 |  |
| Energy intake (kcal/d) |  | 2152.8 ± 584.2 | 2179.6 ± 826.5 | <.001 |
| **Health status factors** | |  |  |  |
| Heart disease | No | 91.4 | 88.2 | <.001 |
|  | Yes | 8.6 | 11.8 |  |
| Hypertension | No | 62.0 | 53.5 | <.001 |
|  | Yes | 38.0 | 46.5 |  |
| Type 2 diabetes | No | 1.6 ± 0.4 | 1.5 ± 0.5 | <.001 |
|  | Yes | 26.5 ± 4.2 | 27.1 ± 4.8 |  |
| HDL cholesterol (mmol/l) |  | 91.2 | 86.5 | <.001 |
| Body Mass Index (kg/m²) |  | 8.9 | 13.5 | <.001 |
| Cognitive impairment | No | 87.2 | 75.0 | <.001 |
|  | Yes | 12.8 | 25.0 |  |
| **Diet quality scores** |  |  |  |  |
| AHEI-2010 ^a^ | | 58.5 ± 10.9 | 56.9 ± 10.8 | 0.79 |
| DASH ^b^ | | 24.1 ± 4.8 | 23.6 ± 4.8 | 0.75 |
| MDS^c^ | | 4.6 ± 1.7 | 4.5 ± 1.6 | 0.39 |
| **Depressive symptoms** | |  |  |  |
| Number of depressive | 0 | 71.9 | 66.0 | <.001 |
| symptoms episodes | 1 | 14.8 | 14.2 |  |
|  | 2 | 13.3 | 19.8 |  |

Values are percentage or means ± standard deviation

^a^ AHEI-2010: Alternate Healthy Eating Index of 2010

^b^ DASH: Dietary Approach to Stop Hypertension

^c^ tMDS: transformed Mediterranean Diet Score

^d^ p-values are based on X² test or t-test

**Supplementary Table B.** Comparison of Whitehall II participants’ characteristics as a function of the 3 diet scores

| **Characteristics of participants** | | **Diet quality scores (n=4949)** | | | | | |
| --- | --- | --- | --- | --- | --- | --- | --- |
|  |  | **AHEI-2010 ^a^** | | **DASH ^b^** | | **tMDS ^c^** | |
|  | | **Mean ± SD** | **p ^d^** | **Mean ± SD** | **p ^d^** | **Mean ± SD** | **p ^d^** |
| **Socio-demographic factors** | |  |  |  |  |  |  |
| Sex | Men | 58.3 ± 10.9 | 0.10 | 24.2 ± 4.8 | 0.02 | 4.6 ± 1.6 | 0.02 |
|  | Women | 58.9 ± 11.0 |  | 23.9 ± 4.7 |  | 4.5 ± 1.7 |  |
| Age (years) |  | 0.036 | 0.01 | 0.063 | <.001 | 0.026 | 0.07 |
| Ethnicity | White | 58.2 ± 10.9 | <.001 | 24.0 ± 4.8 | <.001 | 4.5 ± 1.7 | <.001 |
|  | South Asian | 64.4 ± 10.6 |  | 27.5 ± 4.6 |  | 5.1 ± 1.5 |  |
|  | Black | 61.8 ± 11.1 |  | 25.8 ± 4.5 |  | 5.0 ± 1.5 |  |
| Marital Status | Living alone | 57.5 ± 11.7 | <.001 | 23.6 ± 4.9 | <.001 | 4.3 ± 1.7 | <.001 |
|  | Married | 58.8 ± 10.7 |  | 24.3 ± 4.8 |  | 4.6 ± 1.6 |  |
| Socio-economic status | High | 58.8 ± 10.6 | <.001 | 24.6 ± 4.6 | <.001 | 4.7 ± 1.6 | <.001 |
|  | Intermediate | 58.4 ± 11.2 |  | 23.9 ± 5.0 |  | 4.5 ± 1.7 |  |
|  | Low | 56.9 ± 11.2 |  | 23.0 ± 4.9 |  | 4.2 ± 1.7 |  |
| **Health behaviour factors** | |  |  |  |  |  |  |
| Smoking habits | Never/ex | 58.9 ± 10.8 | <.001 | 24.3 ± 4.8 | <.001 | 4.6 ± 1.6 | <.001 |
|  | Smoking | 53.6 ± 11.4 |  | 21.8 ± 4.8 |  | 4.1 ± 1.6 |  |
| Alcohol intake | No | 56.3 ± 11.0 | <.001 | 23.7 ± 5.3 | <.001 | 4.1 ± 1.6 | <.001 |
|  | Moderate | 60.2 ± 10.7 |  | 24.4 ± 4.7 |  | 4.6 ± 1.7 |  |
|  | Heavy | 54.6 ± 10.3 |  | 23.7 ± 4.6 |  | 4.7 ± 1.6 |  |
| Physical activity | Inactive | 57.1 ± 11.3 | <.001 | 23.6 ± 4.9 | <.001 | 4.4 ± 1.6 | <.001 |
|  | Moderate | 58.1 ± 10.9 |  | 23.8 ± 4.7 |  | 4.5 ± 1.7 |  |
|  | Active | 59.1 ± 10.7 |  | 24.4 ± 4.8 |  | 4.7 ± 1.7 |  |
| Total energy intake (kcal/d) |  | 0.086 | <.001 | 0.008 | 0.54 | 0.177 | <.001 |
| **Health status factors** | |  |  |  |  |  |  |
| Heart disease | No | 58.5 ± 10.9 | 0.26 | 24.1 ± 4.8 | 0.48 | 4.6 ± 1.7 | 0.13 |
|  | Yes | 57.9 ± 10.8 |  | 24.3 ± 5.0 |  | 4.7 ± 1.6 |  |
| Hypertension | No | 58.8 ± 10.8 | 0.02 | 24.2 ± 4.8 | 0.32 | 4.5 ± 1.6 | 0.15 |
|  | Yes | 58.0 ± 11.1 |  | 24.1 ± 4.8 |  | 4.6 ± 1.7 |  |
| Type 2 diabetes | No | 58.5 ± 10.9 | 0.41 | 24.2 ± 4.8 | 0.39 | 4.6 ± 1.7 | 0.68 |
|  | Yes | 58.1 ± 10.7 |  | 24.0 ± 4.7 |  | 4.5 ± 1.6 |  |
| HDL cholesterol (mmol/l) |  | 0.058 | <.001 | 0.067 | <.001 | 0.073 | <.001 |
| Body Mass Index (kg/m²) |  | -0.134 | <.001 | -0.141 | <.001 | -0.071 | <.001 |
| Cognitive impairment | No | 58.4 ± 10.9 | 0.35 | 24.1 ± 4.8 | 0.14 | 4.6 ± 1.6 | 0.52 |
|  | Yes | 58.9 ± 11.0 |  | 24.4 ± 4.8 |  | 4.5 ± 1.7 |  |

Values are means ± standard deviation

^a^ AHEI-2010: Alternate Healthy Eating Index of 2010

^b^ DASH: Dietary Approach to Stop Hypertension

^c^ tMDS: transformed Mediterranean Diet Score

^d^ p-values are based on X² test or ANOVA test

**Supplementary Table C.** Odds ratios (95% confidence intervals) for the association between 11-year change in DASH ^a^ score between 1991/1993 and 2002/2004 and the subsequent recurrent depressive symptoms (DepS) over 13 years of follow-up

| **11-year change in DASH ^a^** | **n** | **OR** | **95%CI** |
| --- | --- | --- | --- |
| Maintaining a high DASH ^a^ score | 1584 | 1.01 | 0.82-1.24 |
| vs. maintaining a low DASH ^a^ score | 1651 | 1 | ref. |
|  |  |  |  |
| Improving DASH ^a^ score | 788 | 0.94 | 0.73-1.27 |
| vs. maintaining a low DASH ^a^ score | 1651 | 1 | ref. |
|  |  |  |  |
| Decreasing DASH ^a^ score | 681 | 1.10 | 0.84-1.44 |
| vs. maintaining a high DASH ^a^ score | 1584 | 1 | ref. |
| ^a^ DASH: Dietary Approach to Stop Hypertension  Models were adjusted for age, sex, ethnicity, marital status, socio-economic status, smoking habits, alcohol intake, physical activity, total energy intake, heart diseases, hypertension, type 2 diabetes, HDL cholesterol, Body Mass Index, cognitive impairment  Maintaining a high score : both scores of 1991/94 and 2002/04 ≥ median score (24)  Maintaining a low score : both scores of 1991/94 and 2002/04 < median score (24)  Improving score : score of 1991/94 < median score (24) and score of 2002/04 ≥ median score (24)  Decreasing score : score of 1991/94 ≥ median score (24) and score of 2002/04 < median score (24) | | | |

**Supplementary Table D.** Odds ratios (95% confidence intervals) for the association between 11-year change in tMDS ^a^ score between 1991/1993 and 2002/2004 and the subsequent recurrent depressive symptoms (DepS) over 13 years of follow-up

| **11-year change in tDMS ^a^** | **n** | **OR** | **95%CI** |
| --- | --- | --- | --- |
| Maintaining a high tDMS ^a^ score | 1584 | 0.86 | 0.66-1.13 |
| vs. maintaining a low tDMS ^a^ score | 1651 | 1 | ref. |
|  |  |  |  |
| Improving tDMS ^a^ score | 788 | 0.83 | 0.64-1.07 |
| vs. maintaining a low tDMS ^a^ score | 1651 | 1 | ref. |
|  |  |  |  |
| Decreasing tDMS ^a^ score | 681 | 1.14 | 0.83-1.57 |
| vs. maintaining a high tDMS ^a^ score | 1584 | 1 | ref. |
| ^a^ tDMS: transformed Mediterranean Diet Score  Models were adjusted for age, sex, ethnicity, marital status, socio-economic status, smoking habits, physical activity, total energy intake, heart diseases, hypertension, type 2 diabetes, HDL cholesterol, Body Mass Index, cognitive impairment  Maintaining a high score : both scores of 1991/94 and 2002/04 ≥ median score (5)  Maintaining a low score : both scores of 1991/94 and 2002/04 < median score (5)  Improving score : score of 1991/94 < median score (5) and score of 2002/04 ≥ median score (5)  Decreasing score : score of 1991/94 ≥ median score (5) and score of 2002/04 < median score (5) | | | |
